# Rotation-invariance is essential for accurate detection of spatially variable genes in spatial transcriptomics

**DOI:** 10.1038/s41467-025-62574-4

**Published:** 2025-08-02

**Authors:** Haohao Su, Yuehua Cui

**Affiliations:** Department of Statistics and Probability, Michigan State University, East Lansing, MI USA

**Keywords:** Statistical methods, Computational models

## Abstract

In spatial transcriptomics, tissue samples are randomly positioned. Rotation-sensitive methods can lead to unreliable spatially variable gene (SVG) detection. We highlight their inherent technical pitfalls and discuss strategies for rotation-invariant methods, enhancing the robustness of SVG detection.

One of the most significant tasks in analyzing spatial transcriptomics data is to efficiently and accurately detect spatially variable genes (SVGs). These genes exhibit specific, non-random spatial patterns across tissue sections, reflecting their significant roles in understanding the spatial heterogeneity in gene expression. Detecting SVGs is essential to reveal molecular mechanisms underlying tissue organization and to better understand biological processes controlling tissue functionality and pathology^1,2^. To address this challenge, numerous computational approaches have been developed, employing advanced statistical, machine learning, and bioinformatics techniques^3^. These methods are intended to enhance SVG detection, interpretability, and performance in large-scale spatial transcriptomics data.

One major limitation of many existing SVG detection methods is that the results are not invariant under different rotations of spatial coordinates. This implies that the results of SVG detection can be largely dependent on the orientation of the tissue sample during analysis. We know that tissue sections are often positioned arbitrarily during sample preparation, resulting in spatial coordinates that differ based on how a sample was positioned. Many methods cannot cope with such variations, which leads to unreliable results, such as high false positives or negatives in SVG detection. This limitation is particularly worsened in the integrative analysis of multi-slice spatial transcriptomics data, where tissue slices are often misaligned (see Fig. 1), thus significantly hindering seamless integration and comparative evaluation across different tissue sections. Ensuring rotation invariance is therefore pivotal to achieving a robust and reliable analysis, especially when analyzing multiple tissue sections of varying orientations.

**Fig. 1:**
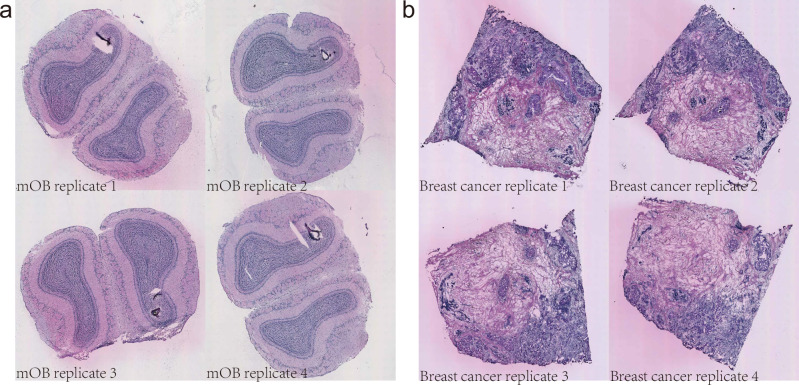
Hematoxylin and eosin-stained brightfield images of mouse olfactory bulb tissue and human breast cancer biopsy^9^. Reproduced and modified from Ståhl et al., *Science*, DOI: 10.1126/science.aaf2403, 2016, AAAS. The original images are from https://www.spatialresearch.org. **a** H& E images of mouse olfactory bulb tissue replicate samples 1, 2, 3, and 4 from ID 1000L2. **b** Hematoxylin and eosin-stained brightfield images of human breast cancer biopsy replicate samples 1, 2, 3, and 4 from ID 1000L3. The variability in tissue orientation and positioning during sample preparation highlights the need for rotation-invariant statistical methods in spatial transcriptomics.

In our comprehensive review of existing SVG detection methods, we identified three prevalent methodological misuses that contribute to the serious issue of rotation variance, compromising the validity of SVG detection (see Fig. 2 for an overview of the three misuses). These misuses are due to inappropriate handling of spatial coordinates in statistical models, leading to flawed assumptions and unreliable outcomes. By synthesizing these findings, we emphasize the need for methodological refinements to effectively address these limitations.

**Fig. 2:**
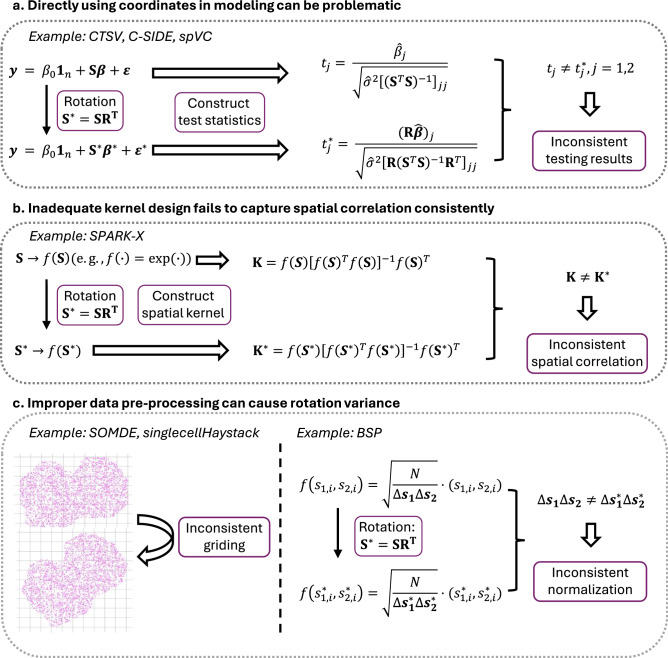
Three prevalent methodological misuses result in inconsistent results due to rotation variance. **a** Directly using coordinates in modeling; **b** inadequate kernel design; **c** improper data pre-processing. Here, ${{{\bf{S}}}}={({{{{\bf{s}}}}}_{1},{{{{\bf{s}}}}}_{2})}_{n\times 2}$ is the spatial coordinate matrix of *n* spots. After applying the spatial rotation, the new spatial coordinate matrix is given as S^*^ = **SR**^*T*^, where **R** is an orthogonal rotation matrix satisfying **R**^*T*^**R** = **RR**^*T*^ = **I**.

## Directly using coordinates in modeling can be problematic

One of the most prominent misuses is when certain methods model spatial coordinates as fixed effects, often accompanied by various transformations or basis expansions in regression models. These models subsequently rely on statistical hypothesis testing of these fixed effects to identify SVGs, including cell-type-specific SVGs (ctSVGs). For instance, CTSV^4^ applies a zero-inflated negative binomial regression model, treating the two spatial coordinates (*s*_1_, *s*_2_) or their transformation (*h*(*s*_1_), *h*(*s*_2_)) as regression covariates, followed by the claim of ctSVG by testing the significance of their associated fixed effect regression coefficients *β*_1_ and *β*_2_. A gene is considered ctSVGs if at least one of these coefficients is statistically non-zero. Similarly, C-SIDE^5^ utilizes a Poisson regression model, incorporating basis expansions of spatial coordinates to model spatial gene patterns. It uses *L* smooth basis functions, whose linear combinations approximate the overall spatial expression pattern. Statistical significance is assessed via two-sided z-tests for each basis function, and the smallest adjusted *p*-value is used to assess the statistical significance. In contrast, spVC^6^ adopts a two-stage Poisson regression approach to identify ctSVGs. In the first stage, it models *K* ctSVGs non-spatial effects and tests their significance to claim SVGs, together with testing a residual spatial random effect. In their second stage, a full model incorporates *K* additional ctSVGs spatial effects, approximated via bivariate penalized spline techniques, to identify ctSVGs. The bivariate spline expansions are based on spatial coordinates and are treated as fixed effects in the estimation and testing of ctSVG, thus exhibiting the rotation invariance issue (see the supplemental file in Su et al.^7^ for a comprehensive evaluation of these methods violating the rotation-invariant property).

In summary, these methods share a fundamental limitation: their statistical tests rely on rotation-variant fixed-effect modeling of spatial coordinates. Consequently, the testing results change when the coordinate system is altered, including through rotation (see the supplemental file for more details).

## Inadequate kernel design fails to capture spatial correlation consistently

Common approaches for SVG detection involve constructing similarity kernel matrices to capture spatial correlation, modeling spatial random effects and then performing hypothesis tests on the corresponding variance components.

However, we noticed that some methods use rotation-variant techniques to construct spatial similarity matrices, leading to potential inconsistencies in statistical inference. For instance, methods that use projection matrices of kernel-transformed spatial coordinates to measure spatial similarity define relationships based on specific coordinate axes, making their results sensitive to rotation (see the supplemental file for more details). Similarly, linear kernels, relying on direct inner products of spatial coordinates, introduce dependencies on the absolute orientation of the coordinate system. As a result, their testing results can change when the coordinate system is rotated, raising concerns about robustness and reproducibility in SVG detection.

**Fig. 3:**
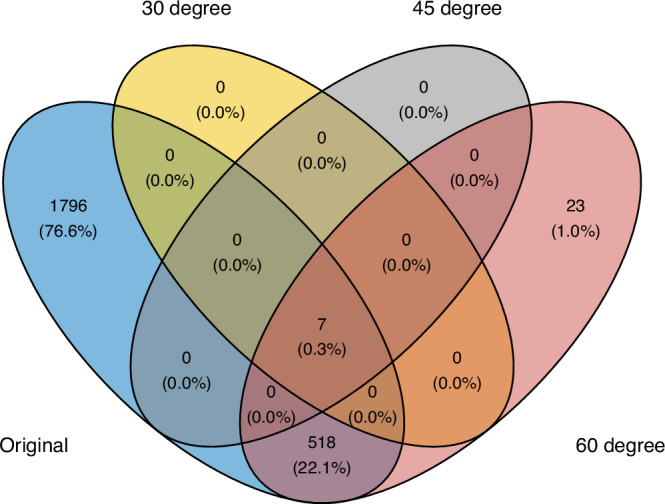
Rotation-variant method yields inconsistent SVGs under different coordinate rotations. The Venn diagram shows the logical relationship between sets of genes identified by SPARK-X^8^ across the original spatial pattern and patterns rotated by 30^∘^, 45^∘^, and 60^∘^ of mouse olfactory bulb (mOB)^9^ tissue replicate #8. Under the original pattern, 2321 genes are identified as SVGs by SPARK-X. However, this number drops sharply to 548 when the tissue is rotated by 60^∘^. Even more strikingly, only seven genes are consistently identified across the 30^∘^ and 45^∘^ rotations. Source data are provided as a Source Data file.

SPARK-X^8^ exemplifies this issue. It constructs a similarity matrix for gene expression and another for spatial patterns using projection matrices, then tests whether these two similarity matrices are independent to determine whether a gene is an SVG. Specifically, the spatial similarity matrix is defined as ${{{\bf{K}}}}={{{\bf{S}}}}^{\prime} ({({{{\bf{S}}}}^{\prime} )}^{T}{{{\bf{S}}}}^{\prime} )^{-1}{{{{\bf{S}}}}}^{{\prime} T}$, where ${{{{\bf{S}}}}}^{{\prime} }$ is the spatial coordinate matrix of dimension *n* × 2 after some transformation such as exponential or cosine. We showed in the supplemental file that such a projection-based similarity matrix maintains the spatial rotation-invariant property if ${{{{\bf{S}}}}}^{{\prime} }$ it is based on the original coordinates without transformation. However, this property is violated when ${{{{\bf{S}}}}}^{{\prime} }$ it is obtained via exponential or cosine transformation of the original coordinates as proposed by SPARK-X. In this case, the testing outcomes of SPARK-X can change when the coordinate system is rotated, compromising the method’s validity and reliability. To illustrate the issue, we applied SPARK-X^8^ to the mouse olfactory bulb tissue replicate #8^9^ under different rotations. Figure 3 shows the inconsistent SVG detection results under different rotations.

Similarly, SMASH^10^ modifies the SPARK-X framework by incorporating two additional alternatives, distance-based Gaussian and cosine kernels, to construct the spatial similarity matrix **K**. These alternatives are rotation-invariant, since they rely solely on relative distances. However, the inclusion of the original projection matrix, which is derived from kernel-transformed spatial coordinates, retains the flaw, making SMASH susceptible to rotation-induced inconsistencies in statistical inference.

## Improper data pre-processing can cause rotation variance

In addition to inappropriate modeling approaches, certain preprocessing procedures can also introduce rotation-variance issues, possibly propagating through the analysis pipeline and impacting the reliability of SVG detection.

One such issue arises in methods that discretize spatial coordinates by dividing the tissue slice into a fixed grid structure along both axes. This type of preprocessing is employed by methods such as singlecellHaystack^11^ and SOMDE^12^, where spatial locations are assigned to grid cells before performing downstream statistical analyses.

The main issue is that the coverage of each grid cell over the tissue is not preserved under rotation. When the coordinate system alters, the boundaries of these predefined grid cells shift relative to the spatial locations of the observed data points, causing inconsistencies in how cells or spots are assigned to different spatial bins. As a result, spatial patterns that were initially captured within specific grid structures may become distorted, potentially leading to different statistical conclusions when the same dataset is analyzed under different orientations.

This rotation-induced variation can significantly affect the modeling and hypothesis-testing steps that follow. For instance, in singlecellHaystack^11^, spatial enrichment is assessed by comparing the density of observed spatial locations within grid cells against a null distribution. If the grid structure changes with rotation, the spatial density estimates may vary accordingly, leading to inconsistent enrichment results. Similarly, in SOMDE^12^, which applies self-organizing maps to discretized spatial domains before conducting variance decomposition, the altered grid structure can impact how spatial dependencies are learned, ultimately affecting SVG detection.

While grid-based preprocessing improves scalability, its sensitivity to rotation can introduce biases, leading to inconsistent SVG detection across different coordinate representations. This highlights the importance of adopting rotation-invariant preprocessing techniques to ensure robustness and reproducibility in SVG detection.

Another source of rotation variance comes from coordinate normalization procedures used in some methods. In particular, BSP^13^, a granularity-based approach for SVG detection, performs normalization of spatial coordinates based on spot density. Specifically, the coordinates along each axis are scaled by an estimated density, which is computed as the total number of spots divided by the sample area. However, the sample area in BSP is defined as the product Δ**s**_1_Δ**s**_2_, where $\Delta {{{{\bf{s}}}}}_{j}=\max ({{{{\bf{s}}}}}_{j})-\min ({{{{\bf{s}}}}}_{j})$ for *j* = 1, 2, corresponding to the range of spatial coordinates along each axis. This definition assumes a rectangular bounding box aligned with the coordinate axes, which is sensitive to rotation. If the tissue sample is not approximately circular, a rotation of the coordinate system can significantly alter the values of Δ**s**_1_ and Δ**s**_2_, and hence the computed area and normalization factors. As a result, the normalized coordinates and the downstream SVG detection may vary with rotation.

## Strategies to ensure rotation-invariant SVG detection

To address the three methodological misuses resulting in rotation variance discussed above, we provide several suggestions for designing SVG detection methods that are robust to rotation.

First, when fitting fixed-effect models to capture spatial variation, it is advisable to avoid using spatial coordinates or their transformations as covariates. A more robust strategy is to construct predictors based on relative distances, which are inherently invariant to rotation. For example, Niche-DE^14^ encodes spatial context by computing a distance-based, kernel-smoothed density for each anchor cell, effectively summarizing locally spatial information. Since this method relies exclusively on pairwise distances, the resulting fixed-effect modeling is rotation-invariant by design.

Second, when modeling spatial correlation through similarity kernels, only distance-based kernel functions should be used. When a distance-based kernel function, such as a distance-based Gaussian kernel, a distance-based cosine kernel, or a Matérn-class kernel, is used to define spatial similarity, the resulting statistical tests remain invariant under spatial coordinate transformations, including rotation (see the supplemental file for more details). This invariance arises because distance-based kernels depend solely on pairwise distances between spatial locations, which are preserved regardless of rotation. Methods like SPARK^15^ and nnSVG^16^ exemplify this approach by adopting distance-based kernels to model spatial correlations.

Third, to avoid the rotation sensitivity introduced by grid-based preprocessing steps, strategies relying on relative positioning should be considered. For example, aggregation schemes based on k-nearest neighbors offer a rotation-invariant alternative, as the neighborhood structure defined by pairwise distances remains unchanged under rotation. Such methods can preserve spatial resolution while maintaining geometric robustness.

## Conclusion

Accurate identification of SVGs is crucial for understanding the spatial organization of gene expression. Our evaluation highlights a critical limitation in many existing SVG detection methods: their sensitivity to tissue positioning during sample preparation. Through the analysis of common methodological misuses, we demonstrated how inappropriate modeling of spatial coordinates can lead to unreliable and inconsistent SVG results. We show that leveraging relative distances provides a more robust framework for capturing spatial patterns while maintaining invariance to tissue rotations. Additionally, improper preprocessing steps can exacerbate rotation-induced variability, complicating the detection of true spatial expression patterns. Addressing the methodological pitfalls is essential to improving the reliability and interpretability of SVG detection. Thus, by adopting methodological refinements and incorporating rotation-invariant strategies, researchers can improve the accuracy and reproducibility of SVG detection, ultimately advancing our understanding of spatial gene expression and its biological significance.

## Supplementary information

Supplementary Information

## Source data

Source Data

## Data Availability

The mouse olfactory bulb and human breast cancer image data^9^ shown in Fig. 1 can be found at https://www.spatialresearch.org. Source data are provided with this paper.
