# Supplementary material for: Rotation-invariance is essential for accurate detection of spatially variable genes in spatial transcriptomics
Source: Nat Commun. 2025 Aug 2;16:7122. doi: 10.1038/s41467-025-62574-4 (PMC12318016; doi:10.1038/s41467-025-62574-4)
Supplement: Supplementary file 1 — Supplementary Information [file 41467_2025_62574_MOESM1_ESM.pdf]

Supplemental file for "Rotation-Invariance is Essential for Accurate Detection of Spatially Variable Genes in Spatial Transcriptomics"

Haohao Su[1] and Yuehua Cui[1*]

[1]Department of Statistics and Probability, Michigan State University, East Lansing, 48824, MI, USA.

*Corresponding author(s). E-mail(s): cuiy@msu.edu;

Fixed-effect statistics are not spatial rotation-invariant

Suppose we have spatial transcriptomics expression data for q genes from n spots (or pixels) of a 2D tissue, with their spatial locations denoted as $\mathbf{S} = (s_{i1}, s_{i2})_{n\times 2}$, $i = 1, \cdots, n$. For one specific gene, the original gene expression count data of n spots are collected and normalized through various methods to yield continuous gene expression data, denoted as $\mathbf{y} = (y_1, \cdots, y_n)^T$.

Here, we first demonstrate why spatial coordinates, including the transformations and basis expansions, are not suitable to work as fixed effects within regression models designed to characterize gene expression patterns. Without loss of generality, we assume the following simplified model,

$$\mathbf{y} = \beta_0 \mathbf{1}_n + \beta_1 h(\mathbf{s}_1) + \beta_2 h(\mathbf{s}_2) + \boldsymbol{\varepsilon}, \tag{1}$$

where $\mathbf{1}_n$ is an n-dimensional vector of ones; $\mathbf{s}_j = (s_{1j}, \cdots, s_{nj})^T$, $j = 1, 2$ is the j-th column of spatial coordinate matrix $\mathbf{S}$; $h(\cdot)$ is a function that implements various spatial transformation which can be an identity or exponential function; $\beta_0, \beta_1, \beta_2$ are regression coefficients and $\boldsymbol{\varepsilon} \sim \text{MVN}(\mathbf{0}_n, \sigma^2 \mathbf{I}_n)$ is the vector of residual error. CTSV[1] implements this model for ctSVG detection.

Taking $h(s) = s$ as an identity function as an example, in matrix form, model (1) can be written as

$$\mathbf{y} = \beta_0 \mathbf{1}_n + \mathbf{S}\boldsymbol{\beta} + \boldsymbol{\varepsilon}, \tag{2}$$

where $\mathbf{S} = [\mathbf{s}_1, \mathbf{s}_2]$ is an $n \times 2$ matrix, and $\boldsymbol{\beta} = (\beta_1, \beta_2)^T$. Furthermore, we can rewrite model (2) as

$$\tilde{\mathbf{y}} = \mathbf{S}\boldsymbol{\beta} + \boldsymbol{\varepsilon}, \tag{3}$$

where $\tilde{\mathbf{y}} = \mathbf{y} - \beta_0 \mathbf{1}_n$.

After applying the spatial rotation, the new spatial coordinate matrix is given as

$$\mathbf{S}^* = \mathbf{S}\mathbf{R}^T, \tag{4}$$

where $\mathbf{R}$ is a rotation matrix defined as

$$\mathbf{R} = \begin{bmatrix} \cos(\frac{\theta}{180}\pi) & -\sin(\frac{\theta}{180}\pi) \\ \sin(\frac{\theta}{180}\pi) & \cos(\frac{\theta}{180}\pi) \end{bmatrix}. \tag{5}$$

The matrix $\mathbf{R}$ is an orthogonal matrix, satisfying $\mathbf{R}^T\mathbf{R} = \mathbf{I}_2$, where $\mathbf{I}_2$ is a 2×2 identity matrix. The new spatial coordinates $\mathbf{S}^*$ implies that a tissue slice is rotated by an angle of θ. Let the columns of $\mathbf{S}^*$ be $\mathbf{s}_1^*$ and $\mathbf{s}_2^*$, then $\mathbf{S}^* = [\mathbf{s}_1, \mathbf{s}_2]\mathbf{R}^T = [\mathbf{s}_1^*, \mathbf{s}_2^*]$.

Model (1) with the rotated coordinates becomes

$$\mathbf{y} = \beta_0 \mathbf{1}_n + \beta_1^* \mathbf{s}_1^* + \beta_2^* \mathbf{s}_2^* + \boldsymbol{\varepsilon}, \tag{6}$$

with the new design matrix $\mathbf{X}^* = [\mathbf{1}_n, \mathbf{s}_1^*, \mathbf{s}_2^*]$, and coefficients $\boldsymbol{\beta}^* = (\beta_0, \beta_1^*, \beta_2^*)^T$. Note that a rotation preserves distances and does not introduce any translation (i.e., no shifting of data points). This means the centroid of the data remains the same. Since the intercept represents the expected value of Y when s_1 and s_2 (or s_1^* and s_2^*) are zero, and since rotation does not change the overall mean structure of the data, the intercept estimate remains the same. Therefore, the rotated model (6) can be rewritten as

$$\tilde{\mathbf{y}} = \mathbf{S}^*\boldsymbol{\beta}^* + \boldsymbol{\varepsilon}^*, \tag{7}$$

where $\tilde{\mathbf{y}} = \mathbf{y} - \beta_0 \mathbf{1}_n$ and $\boldsymbol{\beta}^* = (\beta_1^*, \beta_2^*)^T$.

The t-statistic for a coefficient β_j, $j = 1, 2$ in ordinary least squares (OLS) regression is given as

$$t_j = \frac{\hat{\beta}_j}{\mathrm{SE}(\hat{\beta}_j)}, \tag{8}$$

where $\hat{\beta}_j$ is the OLS estimate, and $\mathrm{SE}(\hat{\beta}_j) = \sqrt{\hat{\sigma}^2[\mathbf{C}]_{jj}}$; $\hat{\sigma}^2 = \frac{1}{n-p}\|\tilde{\mathbf{y}} - \mathbf{S}\hat{\boldsymbol{\beta}}\|_2^2$ is the residual variance ($p = 3$ here); and $\mathbf{C} = (\mathbf{S}^T\mathbf{S})^{-1}$.

Next, we compare the t-statistics for β_1 and β_2 in the original model with those for β_1^* and β_2^* in the rotated model. The OLS estimates before and after rotation are

$$\begin{aligned} \hat{\boldsymbol{\beta}} &= (\mathbf{S}^T\mathbf{S})^{-1}\mathbf{S}^T\tilde{\mathbf{y}}, \\ \hat{\boldsymbol{\beta}}^* &= (\mathbf{S}^{*T}\mathbf{S}^*)^{-1}\mathbf{S}^{*T}\tilde{\mathbf{y}} \\ &= (\mathbf{R}\mathbf{S}^T\mathbf{S}\mathbf{R}^T)^{-1}\mathbf{R}\mathbf{S}^T\tilde{\mathbf{y}} \\ &= \mathbf{R}(\mathbf{S}^T\mathbf{S})^{-1}\mathbf{R}^T\mathbf{R}\mathbf{S}^T\tilde{\mathbf{y}} \end{aligned} \tag{9}$$

$$= \mathbf{R}(\mathbf{S}^T\mathbf{S})^{-1}\mathbf{S}^T\tilde{\mathbf{y}} = \mathbf{R}\hat{\boldsymbol{\beta}}, \tag{10}$$

since $\left[\mathbf{R}\mathbf{S}^T\mathbf{S}\mathbf{R}^T\right]\left[\mathbf{R}(\mathbf{S}^T\mathbf{S})^{-1}\mathbf{R}^T\right] = \mathbf{I}_2 \Longrightarrow \left(\mathbf{R}\mathbf{S}^T\mathbf{S}\mathbf{R}^T\right)^{-1} = \mathbf{R}(\mathbf{S}^T\mathbf{S})^{-1}\mathbf{R}^T$.

For β_1 and β_2 in the original model,

$$\hat{\sigma}^2 = \frac{1}{n-p}\|\tilde{\mathbf{y}} - \mathbf{S}\hat{\boldsymbol{\beta}}\|_2^2 \tag{11}$$

and for β_1^* and β_2^* after rotation, the residual variance estimate remains the same, i.e.,

$$\hat{\sigma}^{*2} = \frac{1}{n-p}\|\tilde{\mathbf{y}} - \mathbf{S}^*\hat{\boldsymbol{\beta}}^*\|_2^2 = \frac{1}{n-p}\|\tilde{\mathbf{y}} - \mathbf{S}\mathbf{R}^T\mathbf{R}\hat{\boldsymbol{\beta}}\|_2^2 = \hat{\sigma}^2$$

The estimated covariance matrix of the estimates is

$$\hat{\text{Var}}(\hat{\boldsymbol{\beta}}) = \hat{\sigma}^2(\mathbf{S}^T\mathbf{S})^{-1}, \tag{12}$$

$$\hat{\text{Var}}(\hat{\boldsymbol{\beta}}^*) = \hat{\sigma}^{*2}(\mathbf{S}^{*T}\mathbf{S}^*)^{-1} = \hat{\sigma}^2\mathbf{R}(\mathbf{S}^T\mathbf{S})^{-1}\mathbf{R}^T. \tag{13}$$

Therefore, the t-statistics for β_1 and β_2 in the original model,

$$t_j = \frac{\hat{\beta}_j}{\sqrt{\hat{\sigma}^2[(\mathbf{S}^T\mathbf{S})^{-1}]_{jj}}}, \tag{14}$$

and those for β_1^* and β_2^* in the rotated model,

$$t_j^* = \frac{(\mathbf{R}\hat{\boldsymbol{\beta}})_j}{\sqrt{\hat{\sigma}^2[\mathbf{R}(\mathbf{S}^T\mathbf{S})^{-1}\mathbf{R}^T]_{jj}}}, \tag{15}$$

are not identical, indicating that the testing results are not invariant to rotation. Note that we illustrated the issue of rotation variance using the original spatial coordinates. Other methods, such as those based on spline expansions of spatial coordinates, suffer from similar issues and should therefore be avoided. Su et al.[2] designed a simulation highlighting the issue of rotation variance in methods such as CTSV[1], C-SIDE[3] and spVC[4] for ctSVG detection. This concern equally applies to SVG detection.

Kernel-based methods and choices of rotation-invariant kernel

Kernel-based methods treat spatial gene effect as random and test for the significance of the variance component (e.g., SpatialDE[5] and SPARK[6]). Given a set of spatial coordinates, we can construct a spatial similarity matrix, denoted as $\mathbf{K}$, using distance-based kernel functions. Below, we present three examples of such kernel functions.

Distance-based Gaussian kernel function:

$$K_{\text{Gaussian}}(\mathbf{s}_i, \mathbf{s}_j) = \exp\left\{-\frac{\|\mathbf{s}_i - \mathbf{s}_j\|_2^2}{2\sigma^2}\right\},$$

where $\|\mathbf{s}_i - \mathbf{s}_j\|_2$ represents the Euclidean distance between points $\mathbf{s}_i = (s_{i1}, s_{i2})^T$ and $\mathbf{s}_j = (s_{j1}, s_{j2})^T$, and σ is the bandwidth parameter that determines the kernel's "spread". Smaller σ values imply quick decay of spatial correlation and vice versa.

Distance-based cosine kernel function:

$$K_{\text{cosine}}(\mathbf{s}_i, \mathbf{s}_j) = \cos\left\{2\pi \frac{\|\mathbf{s}_i - \mathbf{s}_j\|_2}{\phi}\right\},$$

where ϕ is the length scale parameter controlling the periodicity of the kernel. A cosine kernel captures spatial periodic pattern of gene expressions.

Matérn family of kernels[7]:

$$K_{\text{Matérn}}(\mathbf{s}_i, \mathbf{s}_j) = \frac{2^{1-\nu}}{\Gamma(\nu)}\left(\sqrt{2\nu}\frac{\|\mathbf{s}_i - \mathbf{s}_j\|_2}{\ell}\right)^{\nu} K_{\nu}\left(\sqrt{2\nu}\frac{\|\mathbf{s}_i - \mathbf{s}_j\|_2}{\ell}\right),$$

where ℓ is the length scale, Γ is the gamma function, K_ν is the modified Bessel function of the second kind, and $\nu > 0$ is the smoothness parameter governing the kernel's differentiability.

When the spatial coordinates are rotated by an angle θ, the transformed coordinates become $\tilde{\mathbf{s}}_i = \mathbf{R}\mathbf{s}_i$ and $\tilde{\mathbf{s}}_j = \mathbf{R}\mathbf{s}_j$, with the rotation matrix $\mathbf{R}$ defined as:

$$\mathbf{R} = \begin{bmatrix} \cos\left(\frac{\theta}{180}\pi\right) & -\sin\left(\frac{\theta}{180}\pi\right) \\ \sin\left(\frac{\theta}{180}\pi\right) & \cos\left(\frac{\theta}{180}\pi\right) \end{bmatrix}.$$

Since $\mathbf{R}$ is an orthogonal matrix, it satisfies $\mathbf{R}^T\mathbf{R} = \mathbf{I}_2$, where $\mathbf{I}_2$ is the 2×2 identity matrix.

After rotation, the Euclidean distance between the i-th and j-th spots is:

$$\begin{aligned} \|\tilde{\mathbf{s}}_i - \tilde{\mathbf{s}}_j\|_2 &= \sqrt{(\tilde{\mathbf{s}}_i - \tilde{\mathbf{s}}_j)^T(\tilde{\mathbf{s}}_i - \tilde{\mathbf{s}}_j)} \\ &= \sqrt{(\mathbf{R}\mathbf{s}_i - \mathbf{R}\mathbf{s}_j)^T(\mathbf{R}\mathbf{s}_i - \mathbf{R}\mathbf{s}_j)} \\ &= \sqrt{(\mathbf{s}_i - \mathbf{s}_j)^T\mathbf{R}^T\mathbf{R}(\mathbf{s}_i - \mathbf{s}_j)} \\ &= \sqrt{(\mathbf{s}_i - \mathbf{s}_j)^T(\mathbf{s}_i - \mathbf{s}_j)} \\ &= \|\mathbf{s}_i - \mathbf{s}_j\|_2. \end{aligned}$$

This calculation shows that the Euclidean distance $\|\mathbf{s}_i - \mathbf{s}_j\|_2$ remains invariant under rotation. As a result, the distance-based kernel matrix $\mathbf{K}$ is unaffected by spatial rotations of any degree.

Next, we explain why the projection matrices used in methods like SPARK-X [8] and SMASH [9] lack this rotational invariance. The spatial similarity matrix defined by a projection matrix is given by:

$$\mathbf{K} = \mathbf{S}'((\mathbf{S}')^T\mathbf{S}')^{-1}\mathbf{S}', \tag{16}$$

where $\mathbf{S}'$ represent the transformed spatial coordinate matrix, such as using the exponential or cosine transformation [8].

After applying a rotation, the new coordinate matrix becomes:

$$\mathbf{S}^* = \mathbf{S}\mathbf{R}^T. \tag{17}$$

If $\mathbf{S}'$ is the original spatial coordinate matrix $\mathbf{S}$, the updated spatial similarity matrix $\mathbf{K}^*$ is then given by:

$$\begin{aligned}
\mathbf{K}^* &= \mathbf{S}^* \left[(\mathbf{S}^*)^T \mathbf{S}^*\right]^{-1} (\mathbf{S}^*)^T \\
&= \mathbf{S}\mathbf{R}^T \left[(\mathbf{S}\mathbf{R}^T)^T \mathbf{S}\mathbf{R}^T\right]^{-1} (\mathbf{S}\mathbf{R}^T)^T \\
&= \mathbf{S}\mathbf{R}^T \left(\mathbf{R}\mathbf{S}^T\mathbf{S}\mathbf{R}^T\right)^{-1} \mathbf{R}\mathbf{S}^T \\
&= \mathbf{S}\mathbf{R}^T\mathbf{R} \left(\mathbf{S}^T\mathbf{S}\right)^{-1} \mathbf{R}^T\mathbf{R}\mathbf{S}^T \\
&= \mathbf{S} \left(\mathbf{S}^T\mathbf{S}\right)^{-1} \mathbf{S}^T,
\end{aligned} \tag{18}$$

which is identical to the original $\mathbf{K}$ prior to rotation. Therefore, the projection-based kernel matrix based on the original coordinates maintains the spatial rotation-invariant property and is safe to apply for SVG detection.

However, if $\mathbf{S}'$ is a non-linear transformation of $\mathbf{S}$, i.e., $\mathbf{S}' = f(\mathbf{S})$, where $f(\cdot)$ is a non-linear function such as exponential or cosine, then:

$$\begin{aligned}
\mathbf{K}^* &= \mathbf{S}^* \left[(\mathbf{S}^*)^T \mathbf{S}^*\right]^{-1} (\mathbf{S}^*)^T \\
&= f(\mathbf{S}\mathbf{R}^T) \left[f(\mathbf{S}\mathbf{R}^T)^T f(\mathbf{S}\mathbf{R}^T)\right]^{-1} f(\mathbf{S}\mathbf{R}^T)^T \neq \mathbf{K},
\end{aligned} \tag{19}$$

since $f(\mathbf{S}\mathbf{R}^T) \neq f(\mathbf{S})\mathbf{R}^T$ for a non-linear $f(\cdot)$.

This illustrates that a projection-based spatial similarity matrix derived from non-linearly transformed coordinates lacks a rotation-invariant property and should be avoided for SVG detection.

Supplementary References

1. Yu, J. & Luo, X. Identification of cell-type-specific spatially variable genes accounting for excess zeros. *Bioinformatics* **38,** 4135–4144 (2022).
2. Su, H., Wu, Y., Chen, B. & Cui, Y. STANCE: a unified statistical model to detect cell-type-specific spatially variable genes in spatial transcriptomics. *Nature Communications* **16,** 1793 (2025).
3. Cable, D. M. *et al.* Cell type-specific inference of differential expression in spatial transcriptomics. *Nature Methods* **19,** 1076–1087 (2022).
4. Yu, S. & Li, W. V. spVC for the detection and interpretation of spatial gene expression variation. *Genome Biology* **25,** 103 (2024).
5. Svensson, V., Teichmann, S. A. & Stegle, O. SpatialDE: identification of spatially variable genes. *Nature Methods* **15,** 343–346 (2018).

6. Sun, S., Zhu, J. & Zhou, X. Statistical analysis of spatial expression patterns for spatially resolved transcriptomic studies. *Nature Methods* **17,** 193–200 (2020).
7. Genton, M. G. Classes of kernels for machine learning: a statistics perspective. *Journal of Machine Learning Research* **2,** 299–312 (2001).
8. Zhu, J., Sun, S. & Zhou, X. SPARK-X: non-parametric modeling enables scalable and robust detection of spatial expression patterns for large spatial transcriptomic studies. *Genome Biology* **22,** 184 (2021).
9. Seal, S., Bitler, B. G. & Ghosh, D. SMASH: Scalable Method for Analyzing Spatial Heterogeneity of genes in spatial transcriptomics data. *PLoS Genetics* **19,** e1010983 (2023).
